# Different brain spatial metabolic patterns characterizing different subtypes of multiple system atrophy

**DOI:** 10.1177/1877718X261434367

**Published:** 2026-04-15

**Authors:** Tiffany Carther-Krone, Jarrad Perron, Chong Sik Lee, Ji Hyun Ko

**Affiliations:** 1 Department of Human Anatomy and Cell Science, Max Rady College of Medicine, University of Manitoba, Winnipeg, MB, Canada; 2 PrairieNeuro Research Centre, Kleysen Institute for Advanced Medicine, Winnipeg Health Science Centre, Winnipeg, MB, Canada; 3 Graduate Program in Biomedical Engineering, Price Faculty of Engineering, University of Manitoba, Winnipeg, MB, Canada; 4 Department of Neurology, Cha University Bundang Medical Center, Seongnam, South Korea

**Keywords:** multiple system atrophy, MSA-Cerebellar, MSA-Parkinson's, metabolic pattern

## Abstract

Multiple system atrophy (MSA) includes parkinsonian (MSA-P) and cerebellar (MSA-C) subtypes. This study characterizes the spatial pattern of brain metabolic PET for MSA subtypes and assesses their relationship to striatal dopamine transporter (DAT) loss, considering sex, age and disease duration. We studied 270 MSA patients using ^18^F-flurodeoxyglucose-PET and scaled subprofile modeling (SSM) to characterize subtype-specific disease-related metabolic patterns and ^18^F-fluoro-propyl-β-CIT-PET to quantify striatal DAT loss. SSM analysis characterized an MSA-P-related metabolic pattern (MSAPRP-Combined), which was created by linear combination of two principal components (PC) that differentiated MSA-P from 16 age-matched controls subsequently named as MSAPRP-PC1 and MSAPRP-PC2. For MSA-C-related pattern (MSACRP), only one PC (MSACRP-PC1) differentiated the MSA-C patients from the same healthy controls, therefore MSACRP-PC1 equaled to MSACRP. MSAPRP-Combined showed hypermetabolism in the pons, the posterior lobe of the cerebellum, the pallidum and in the sensorimotor cortex, and hypometabolism in the putamen, the vermis of the cerebellum, the lateral premotor cortex and the parieto-occipital association regions. MSACRP showed hypometabolism in the cerebellum and putamen. MSAPRP-PC1 was topographically more similar to MSAPRP-Combined while MSAPRP-PC2 was more similar to MSACRP. MSA-P had greater DAT loss than MSA-C and controls. In MSA-P, age correlated with MSAPRP-Combined and DAT binding, while disease duration correlated with DAT. For MSA-P females, MSAPRP-PC2 correlated with disease duration. In MSA-C, MSACRP correlated with disease duration and, in females, with age. These findings suggest that metabolic and dopaminergic relationships with disease progression may differ by sex and age in MSA subtypes.

## Introduction

Multiple system atrophy (MSA) is a progressive, adult-onset neurodegenerative disorder characterized by varying severity of parkinsonian features, cerebellar ataxia, autonomic dysfunction - including urogenital dysfunction - and corticospinal disorders.^1–4^ Classified as an atypical Parkinsonism, it consists of two subtypes: a Parkinsonian variant reflecting underlying predominant nigrostriatal degeneration (MSA-P) and a cerebellar variant associated with predominant olivo-pontocerebellar atrophy (MSA-C).^5^ Currently, there are no disease-modifying therapies for MSA and symptomatic treatments are limited due to a lack of validated, disease-specific biomarkers, complicating clinical trials. To address this, we examined the relationship between a previously characterized MSA-related spatial covariance pattern and presynaptic nigrostriatal dopaminergic dysfunction in both MSA-P and MSA-C patients.

2-[^18^F]fluoro-2-deoxy-D-glucose positron emission tomography (FDG-PET) detects metabolic brain changes by measuring glucose consumption in brain tissue, reflecting neuronal activity and synaptic function and density.^6^ In regions with increased synaptic activity brain metabolism is higher, whereas regions with less activity or those affected by neurodegeneration show lower brain metabolism.^7^ Using FDG-PET images, specific disease related information about spatial pattern of brain synaptic activity can be quantified by applying a scaled subprofile modeling (SSM), a form of principal component analysis (PCA).^8^ This analysis looks for network-level functional abnormalities in neurodegenerative disorders like MSA, allowing for detection of specific disease related spatial metabolic patterns that can serve as a disease biomarker.^9^ In a separate subject group independent of the initial identification cohort the expression of a given disease-related metabolic pattern is quantified prospectively from each individual subject's FDG-PET image using topographic profile rating (TPR) analysis, which can then be compared to previously identified and validated disease related patterns and used for differential diagnosis purposes.

Previous research has both established and validated a distinct disease-related spatial covariance pattern for MSA (MSARP), which is mainly characterized by relatively decreased metabolism in the putamen and the cerebellum,^10^ contrasting with patterns seen in Parkinson's disease (PDRP).^11^ FDG-PET has been effective in distinguishing “look-alike” Parkinsonian diagnoses such as MSA from PD,^12–15^ revealing differing glucose metabolic patterns based on predominant MSA symptoms. While similar increases in MSARP expression overall have been observed in both MSA-P and MSA-C subtypes,^12^ MSA-P show relatively decreased glucose metabolism in the striatum,^16–19^ while MSA-C show relatively decreased glucose metabolism in the cerebellum.^19,20^

F-18 fluorinated-N-3-fluoropropyl-2-b-carboxymethoxy-3-b-(4-iodophenyl)nortropane positron emission topography (FP-CIT-PET) assesses dopaminergic degeneration through dopamine transporter (DAT) density. Previous studies have shown that DAT binding in MSA differs from that of other parkinsonian syndromes,^21^ and that MSA presents significant DAT loss,^22,23^ with MSA-P showing relatively more severe DAT loss in the striatum compared to MSA-C.^24,25^ Understanding the relationship between glucose metabolism and dopaminergic integrity is crucial for developing reliable biomarkers for MSA and its subtypes.

Sex differences have become an important consideration in disease research, with variations in symptoms being noted in MSA such that differences in autonomic and motor symptoms have been reported between males and females with many studies focusing on survival rate.^26–28^ However, it is unclear if sex affects disease-specific biomarkers in MSA. Adjusting for sex-related metabolic changes has shown potential in improving diagnosis of parkinsonism's like MSA,^29^ but further research is needed to confirm whether considering sex-based differences will facilitate early and differential diagnosis of MSA and its subtypes.

Benefiting from a large cohort of MSA patients followed at a single movement disorder clinic, we investigated the effects of age and sex on metabolic covariance patterns relevant to different subtypes of MSA, as well as on striatal dopamine degeneration. We further examined how these subtype-specific metabolic patterns and dopamine loss relate to disease duration, thereby assessing their potential links to disease progression.

## Methods

### Subjects

We retrospectively reviewed 271 MSA patients at Asan Medical Center between March 2004 and December 2018. Inclusion criteria were i) a diagnosis of probable MSA according to second consensus criteria^30^ and ii) completion of FDG-PET, FP-CIT-PET and volumetric T1-weighted MRI. Of these, 140 had MSA-P and 131 had MSA-C. As reference groups, we selected 16 healthy volunteers from the database of normal brain scans maintained at Asan Medical Center (HC1) and 172 healthy controls (HC2) from the Alzheimer's Disease Neuroimaging Initiative (ADNI). All patients underwent metabolic imaging with FDG-PET and dopaminergic imaging with FP-CIT-PET on separate days within a month (the majority of patients were scanned a day apart for two PET scans). The study data collection was approved by the Institutional Review Board of Asan Medical Center, and the data analysis was approved by the Health Research Ethics Board of University of Manitoba. Informed consent was provided by the participants. All participants provided written informed consent for the use of their collected data for research purposes at the time of initial data collection.

### ^18^F-FDG PET imaging and preprocessing

All subjects fasted for at least 6 h prior to imaging. A 5-min transmission scan using a rotating ^68^Ge pin source and a 15-min emission scan was acquired on the ECAT HR+ scanner (Siemens Medical Systems Inc., Hoffman Estate, IL, USA) at the Asan Medical Center, 40 min after injection of 370 MBq of FDG. PET data were processed using an in-house pipeline (*Slapdash*), which was used to standardize all PET imaging data used in the present work. In brief, raw static PET images were coregistered to their T1-weighted anatomical magnetic resonance (MR) images before normalization to the standard MNI template space. These PET images were then masked to remove any extracranial voxels and smoothed using a standard isotropic 8 mm Gaussian filter. Subject MR images were normalized to the same MNI template space through Computational Anatomy Toolbox (CAT12) using default parameters.^31,32^ Extraction of subject-specific putamen and caudate was performed by element-wise multiplication of subject's grey matter tissue image with binary masks of each region.

### Network analysis

Following PET data processing, metabolic brain network analysis was conducted using a voxel-based spatial covariance mapping algorithm called SSM/PCA,^14^ implemented in ScanVp (software freely available online: http://www.feinsteinneuroscience.org at Center for Neuroscience, the Feinstein Institutes for Medical Research, Manhasset, NY). This method extracts covariance data from brain images after removing mean effects across voxels and subjects. After a natural log transformation, group-wise PET data are reduced to a series of independent covariance maps and corresponding subject scores, which reflect the degree of pattern expression for each individual. Disease-specific metabolic patterns were defined by examining orthogonal principal components (PCs) whose expression maximally discriminates between patient and control groups. PCs with a high variance accounted for (vaf) and significant differentiation between patient and control groups are considered for further analysis. In this study, PCs with a vaf > 5% that discriminated between groups using *p* < .05 as the cut-off were identified as part of a disease-related metabolic pattern. For each subgroup significant PCs were examined separately and, if more than one significant PC was found, were linearly combined using logistic regression of subject scores to determine the optimal combination that best separated patients from controls (implemented in ScanVp^33^). Subject scores of this pattern were computed prospectively in the remaining MSA-P and MSA-C patients using a voxel-based topographic profile rating (TPR) algorithm.^34,35^ This was done by taking the dot product each profile image (double-centering after log-transformed) with the network pattern across the brain. Network scores were z-scored against healthy controls (HC1) and comparisons across groups were made to ensure MSARPs were specific to each subgroup.

**Figure 1.**
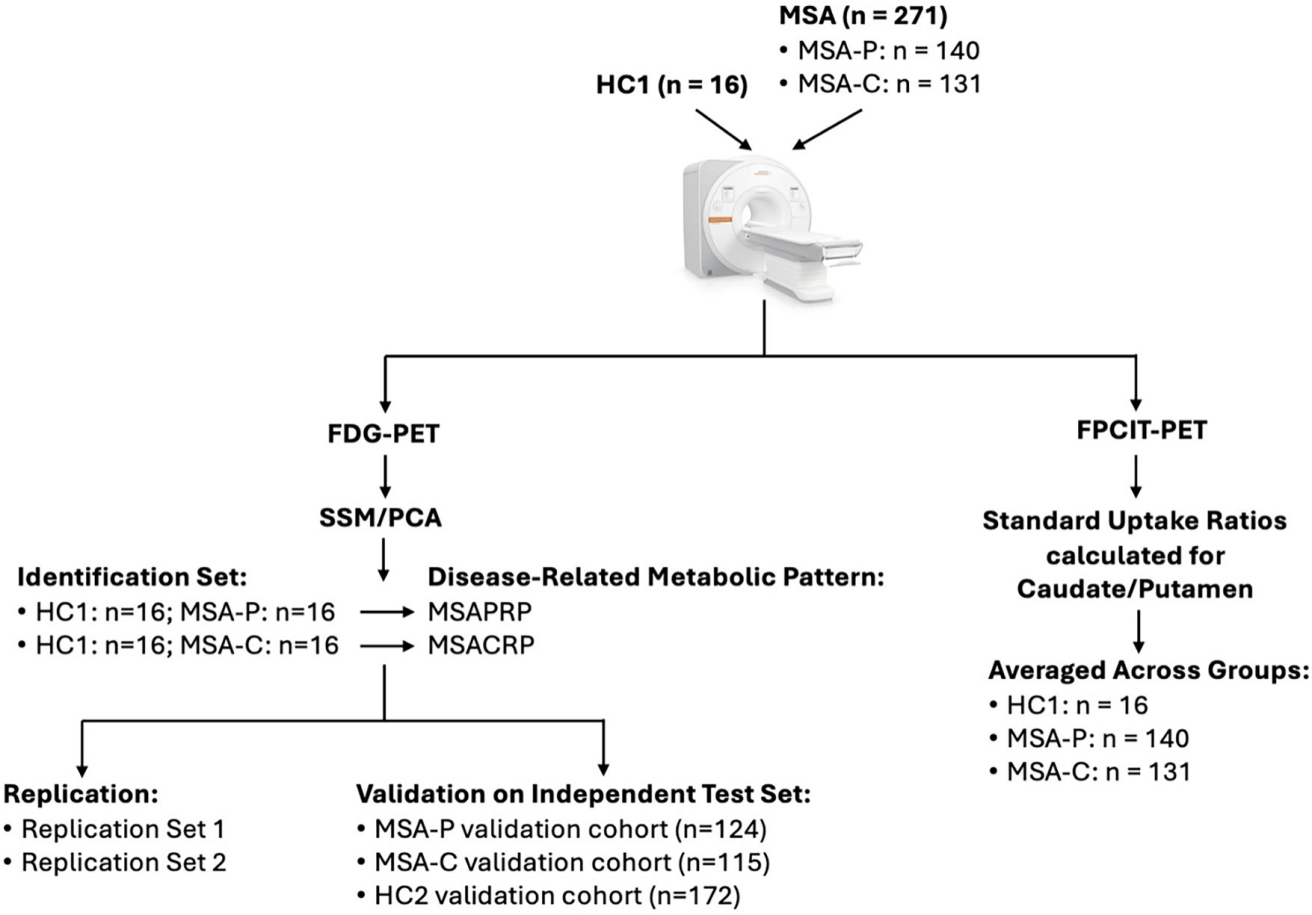
Schematic of methodology. 271 MSA patients (MSA-P: n = 140; MSA-C: n = 131) and 16 healthy controls were scanned using FDG-PET and FP-CIT-PET imaging. Disease-related metabolic patterns were derived using SSM/PCA algorithms. MSA-P and MSA-C specific metabolic patterns were then validated using independent test sets. Reliability of the metabolic patterns were assessed by repeating the SSM/PCA technique using a new age- and sex-matched MSA-P and MSA-C identification set (the first replication set) and an identification set consisting of randomly selected MSA-P and MSA-C patients (the second replication set). Differences in DAT binding was assessed by comparing standard uptake ratios calculated for the caudate and putamen (using the occipital lobe as the reference region) across MSA-P, MSA-C, and healthy control groups.

**Pattern identification and disease discrimination**. MSA-P (MSAPRP) and MSA-C (MSACRP) patterns were first identified using FDG-PET data from 16 healthy controls (HC1) and either 16 MSA-P (for MSAPRP) or 16 MSA-C (for MSACRP) age- and sex-matched patients, forming the MSA-P and MSA-C identification sets (Figure 1). If multiple MSA patients matched a healthy control in age and sex, the final selection was made using a random number generator. The reliability of this pattern was estimated at each voxel by means of an iterative bootstrapping scheme.^35,36^ This step produced a map of inverse coefficient of variation (ICV) to assess a brain-wide statistic. Network scores of MSA-P and MSA-C were then computed and z-scored using the subject scores of the healthy controls in the identification cohort. ICV maps were threshold at |z| > 1.96 and converted into binary masks using SPMs ImCalc. These binary masks were applied to the topographical patterns found in the SSM/PCA analysis to visualize only the statistically significant region weights of the disease-related patterns (p < 0.05).

**Replication of Topographies**. Reliability was further assessed by repeating SSM/PCA with a new age- and sex-matched MSA-P and MSA-C identification set (the first replication set) and an identification set consisting of randomly selected MSA-P and MSA-C patients (the second replication set; see supplementary material). Topographical similarity tests were performed to compare the PCs generated from the original identification set and those of the replicated identification sets to ensure consistency in pattern expression scores.^37^

**Pattern Identification Across MSA Subtypes.** To evaluate the hypothesized metabolic spectrum between MSA-P and MSA-C, a secondary SSM/PCA analysis was performed using the pooled group of MSA-C and MSA-P. This approach allows identification of spatial covariance patterns that maximally discriminate MSA-P from MSA-C. Subject scores of the resulting pattern were then computed prospectively using the TPR algorithm in the independent MSA-P and MSA-C test groups. Comparisons between identification and test groups were used to confirm pattern stability and subtype specificity.

### Analysis of regional metabolic activity

To aid the understanding of the relevance of glucose metabolic patterns identified by SSM/PCA, more traditional voxel-wise statistical analysis was also performed. Differences in regional metabolic activity between patients and healthy controls were detected at each voxel according to the general linear model (GLM) in SPM software. For each MSA subgroup, a two-sample t-test was performed to characterize metabolic activity in MSA-P or MSA-C who were used in the derivation of the disease-related patterns (the identification groups) compared with healthy controls. The contrasts for the decreased and increased metabolism were set as [−1, 1] and [−1, 1]. To evaluate significant differences, a threshold of *p* < .001 (uncorrected) was set over the whole-brain, and clusters were considered significant if they survived at a family-wide error (FWE)-corrected *p* < .05. Resulting SPM maps showing increased or decreased metabolic activity were overlaid on a standard T1-weighted MRI brain template in MRIcron.

### FP-CIT-PET imaging and preprocessing

Image acquisition began 3 h after intravenous injection with 185 MBq FPCIT using Biograph 40 TruePoint PET/CT camera (Siemens Medical Systems, Hoffman Estate, IL, USA). Three-dimensional (3D) emission PET data were acquired for 10 min as described in detail elsewhere.^16^ Following preprocessing similar to FDG-PET, subject-specific regions-of-interest for the putamen, caudate and occipital lobe (reference region) were placed on each normalized FPCIT PET image. Binary masks of each anatomical region from automated anatomical labeling (AAL3) were used to extract specific-tissue ROIs from subject-specific MR images.^38^ These were binarized to avoid exacerbating partial volume effects and then used to extract values from FP-CIT-PET images using element-wise multiplication. Standard uptake ratio (SUR) values were computed by dividing the right/left averaged standard uptake value (SUV) for the caudate and the putamen by the corresponding occipital lobe values.

### Statistical analysis

Group differences in MSAPRP and MSACRP expression were evaluated using a GLM, with age and sex as covariates. Analogous comparisons were made with caudate and putamen FP-CIT-PET SUR values. Pearson correlation coefficients were calculated to correlate pattern expression scores and FP-CIT-PET SUR values with age and disease duration. To determine sex-based differences a GLM was performed using age/disease duration as the dependent variable and sex as the between-subjects factor, with network pattern expression score/DAT binding as the covariate. Significant findings were further explored with separate correlation analyses for males and females. All analyses were considered significant at *p* < .05, using Bonferroni correction for multiple comparisons. Correlation analyses were carried out as two-tailed tests. All statistical analyses were performed using Statistical Package for the Social Sciences (SPSS; IBM Corp., version 27.0, Armonk NY).

## Results

### Demographic differences

Demographic data is summarized in Table 1. MSA-P and MSA-C groups differed in age (t(271) = −4.429, *p* < .001) and disease duration (t(271) = −2.528, *p* = .013), with MSA-C patients being younger on average and having shorter symptom duration. ADNI healthy controls (HC2) also differed significantly in age from healthy controls (HC1) and MSA groups (*p* < .001). No significant differences in age or disease duration were found between identification (n = 16, which matched the 16 HC1) and test (patients who were not selected as the identification set) sets within each subgroup. The MSA-P group had more female participants (*p* = .043), but there were no age or disease duration differences between males and females in any group.

**Table 1.** Demographic comparisons.

	Subjects	Age (years)	Disease duration
Demographic Comparison^a^			
MSA-P	140	63 ± 8.42	2.8 ± 1.82
MSA-C	131	58.99 ± 7.14	2.27 ± 1.69
HC1	16	60.69 ± 8.83	∼
HC2	172	71.69 ± 4.75	∼
p-value		<.001	0.013
Comparison between Identification/Test Sets^b^			
MSA-P			
MSA-P Identification	16	61 ± 8.4	2.5 ± .966
MSA-P Test	124	63.26 ± 8.42	2.84 ± 1.9
p-value		0.314	0.479
MSA-C			
MSA-C Identification	16	58.74 ± 6.84	2.3 ± 1.76
MSA-C Test	115	60.81 ± 9.04	2.06 ± 1.06
p-value		0.278	0.6
Demographic Comparison by Sex^c^			
MSA-P			
Male	58	63.41 ± 8.36	2.54 ± 1.51
Female	82	62.71 ± 8.5	2.99 ± 2.0
p-value	0.043	0.626	0.154
MSA-C			
Male	74	58.7 ± 6.68	2.18 ± 1.49
Female	57	59.37 ± 7.74	2.39 ± 1.92
p-value	0.137	0.599	0.463
HC1			
Male	7	64.86 ± 8.71	∼
Female	9	57.44 ± 7.89	∼
p-value	0.617	0.096	
HC2			
Male	75	72.1 ± 4.533	∼
Female	97	71.38 ± 4.91	∼
p-value	0.093	0.327	

Abbreviations: MSA = multiple system atrophy; MSA-P = MSA-Parkinsonism; MSA-C = MSA-Cerebellar; HC1 = Healthy Control from Asan Medical Center; HC2 = Healthy Controls from ADNI (Alzheimer's disease neuroimaging initiative) database. ^a^For age, p-values reflect analysis of variance; for disease duration, p-values reflect t test. ^b^For age and disease duration, p-values reflect t test. ^c^For subjects, p values reflect the Pearson X^2^ test; for age and disease duration, p values reflect t tests between males and females for each group. Values are means ± SD.

### Metabolic network expression

For the MSA-P group, two disease-related principal components (PCs) were identified (MSAPRP-PC1 and MSAPRP-PC2), which were combined to form an overall MSAPRP (MSAPRP-Combined). For the MSA-C group, one disease-related PC was identified (MSACRP; Supplementary Table S1).

**Figure 2.**
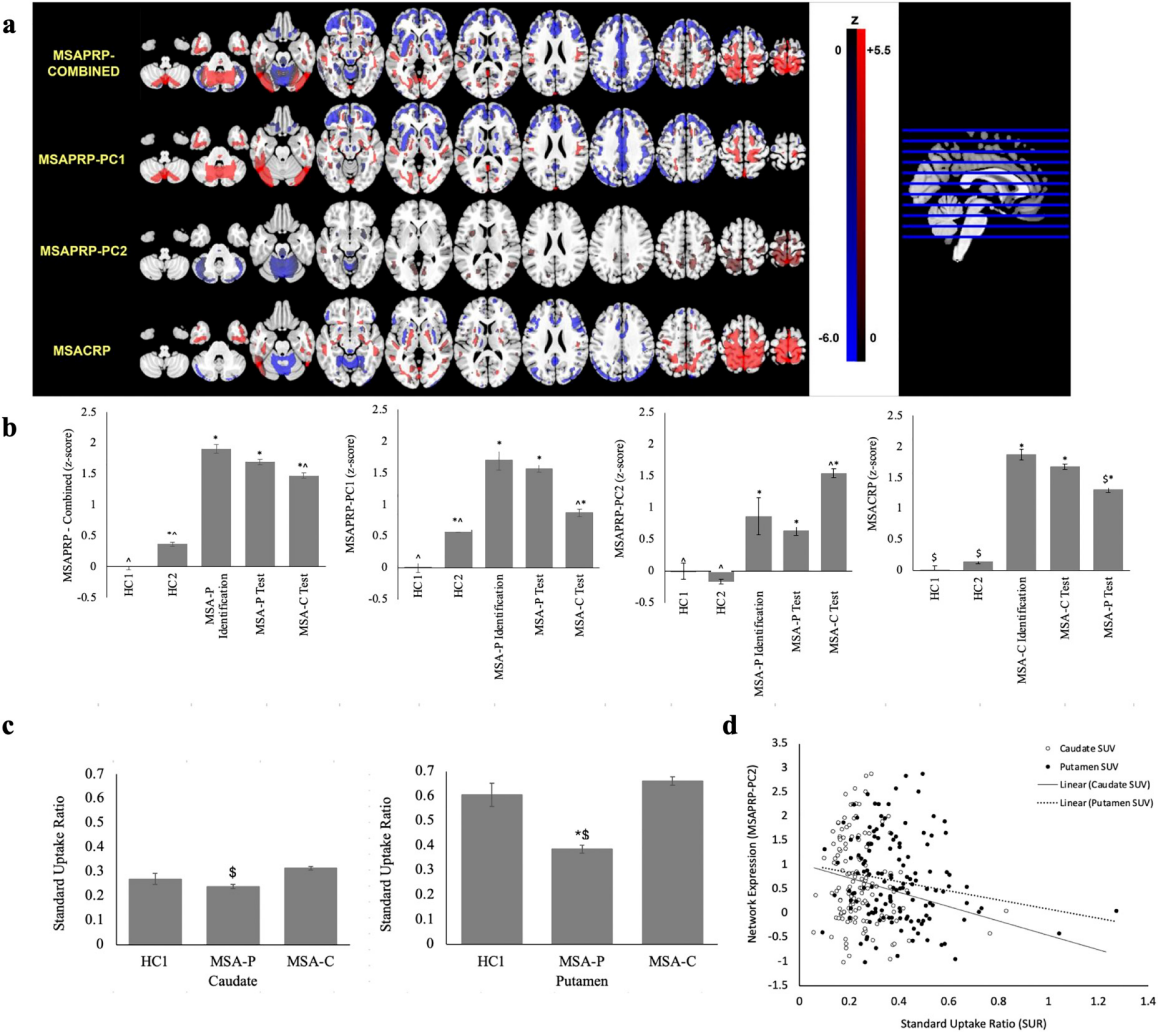
Group comparisons of topography, metabolic expression and dopaminergic integrity. a) Metabolic patterns derived from the SSM/PCA analysis were masked with binary masks created from ICV maps threshold at z = 1.96. MSAPRP-Combined was characterized overall by relatively increased metabolism in the pons, the posterior lobe of the cerebellum, the pallidum and in the sensorimotor cortex, and relatively decreased metabolism in the putamen, the vermis of the cerebellum, the lateral premotor cortex and the parieto-occipital association regions (top row). MSAPRP-PC1 was characterized mainly by relatively decreased metabolism in the putamen (second row), while MSAPRP-PC2 was characterized mainly by relatively decreased metabolism in the cerebellum (third row). The MSACRP was characterized by relatively decreased activity in the cerebellum (bottom row). b) Significant differences in MSARP subject scores were present across both MSA-P and MSA-C subgroups. MSARP scores were significantly elevated in MSA-P and MSA-C groups compared to healthy controls. Significant differences were also found between MSA-P and MSA-C groups for each metabolic pattern. c) Compared to healthy controls, abnormal reductions in putamen DAT binding were present in the MSA-P but not the MSA-C group. Putamen DAT binding was also reduced in the MSA-P group compared to the MSA-C group. Caudate DAT binding was reduced in the MSA-P group compared to the MSA-C group, however, no significant differences were found between healthy controls and the MSA-P group or MSA-C group. d) Significant correlations were found between MSAPRP-PC2 and DAT binding in both the caudate (*r* = −.171, *p* = .043) and putamen (*r* = −.173, *p* = .04) for the MSA-P group. No significant correlations were observed between MSAPRP-Combined or MSAPRP-PC1 and striatal DAT binding in the MSA-P group or between MSACRP and striatal DAT binding in the MSA-C group. *Significantly different from HC1. ^Significantly different from MSA-P groups (*p* < .001). ^$^Significantly different from MSA-C groups (*p* < .001).

Pattern expression scores differed significantly across patient and control groups (Figure 2B). Within each subtype, identification and test groups did not differ, indicating stability of the identified patterns. However, both MSAPRP and MSACRP scores robustly distinguished their respective subtypes from controls, and from each other (Table 2). No significant sex differences in metabolic network expression were found (Supplementary Table S2).

**Table 2.** Group comparisons of metabolic pattern expression scores across MSA subtypes and healthy controls.

Comparison	MSAPRP-Combined	MSAPRP-PC1	MSAPRP-PC2	MSACRP
MSA-P Identification vs.	n.s.	n.s.	n.s.	-
MSA-P Test				
MSA-P Identification vs.	p = .005	p < .001	p = .002	-
MSA-C Test				
MSA-P Test vs.	p = .001	p < .001	p < .001	-
MSA-C Test				
MSA-P Identification vs.	p < .001	p < .001	p < .001	-
HC2				
MSA-P Test vs.	p < .001	p = .006	p < .001	-
HC2				
MSA-C Identification vs.	-	-	-	n.s.
MSA-C Test				
MSA-C vs.	-	-	-	p < .001
MSA-P				
MSA-C Identification vs.	-	-	-	p < .001
HC2				
MSA-C Test vs.	-	-	-	p < .001
HC2				

The spatial covariance pattern for the overall MSA-P network (MSAPRP-Combined) was characterized by relative metabolic increases in the pons, the posterior lobe of the cerebellum, the pallidum and in the sensorimotor cortex, which covaried with metabolic decreases in the caudate, putamen, the vermis of the cerebellum, the lateral premotor cortex and the parieto-occipital association regions. When examined separately, MSAPRP-PC1 was characterized by relative hypermetabolism in the pons and posterior lobe of the cerebellum, the pallidum and in the sensorimotor cortex, along with hypometabolism in the lateral premotor cortex and in parieto-occipital association regions. MSAPRP-PC2 primarily showed relative metabolic reductions in the cerebellar vermis.

As expected, MSAPRP-PC1 and MSARPRP-PC2 were orthogonal to each other (*r* = 0). Nevertheless, all three MSA-P patterns showed spatial overlap with the MSA-C network, with the highest similarity for MSAPRP-Combined (*r* = 0.85, *p* < .001; Table 3). Among the individual PCs, MSAPRP-PC2 (*r* = 0.73, *p* < 0.001) was more similar to MSACRP than MSAPRP-PC1 (*r* = 0.55, *p* = 0.002), consistent with the shared hypometabolism in the cerebellum and putamen (Figure 2A).

**Table 3.** Voxel-wise topographical correlation (r) of original identification set and replication sets. P-values are corrected for auto-correlation.^37^ The replications of the same patterns are bolded**.**

	Original identification set			
Original Identification Set	MSAPRP-Combined	MSAPRP-PC1	MSAPRP-PC2	MSACRP
MSAPRP-Combined	1	.85 (p < .001)	.52 (p < .002)	.85 (p < .001)
MSAPRP-PC1	.85 (p < .001)	1	0 (p = 1)	.55 (p = .002)
MSAPRP-PC2	.52 (p < .002)	0 (p = 1)	1	.73 (p < .001)
MSACRP	.85 (p < .001)	.55 (p = .002)	.73 (p < .001)	1
Replication Sets				
Replication Set 1				
MSAPRP-Combined	.85 (p < .001)	.51 (p = .002)	.86 (p < .001)	.72 (p < .001)
MSAPRP-PC1	.72 (p < .001)	.91 (p < .001)	−.1 (p = .454)	.39 (p = .018)
MSAPRP-PC2	.56 (p = .002)	.19 (p = .190)	.74 (p < .001)	.76 (p < .001)
MSACRP	.61 (p < .001)	.21 (p = .172)	.82 (p < .001)	.84 (p < .001)
Replication Set 2:				
MSAPRP-Combined	.82 (p < .001)	.92 (p < .001)	.38 (p = .018)	.36 (p = .024)
MSAPRP-PC1	.80 (p < .001)	.88 (p < .001)	−.18 (p .245)	.32 (p = .038)
MSAPRP-PC2	.19 (p = .194)	.29 (p = .058)	.76 (p < .001)	.81 (p < .001)
MSACRP	.62 (p < .001)	.37 (p = .01)	.76 (p < .001)	.9 (p < .001)

Replication analyses confirmed these findings (Supplementary Tables S3-S7). Across both MSA-P replication sets, MSAPRP-PC1 again showed relative metabolic increases in the pons and cerebellum paired with decreases in the premotor cortex and parieto-occipital association regions, while MSAPRP-PC2 showed relative reductions in the cerebellum and putamen. Similarly, across both MSA-C replication sets, MSACRP consistently showed relative hypometabolism in the cerebellum and putamen. Voxel-wise correlations^37^ confirmed that the newly-defined MSARPs closely matched the original topographies (Table 3).

The SSM/PCA analysis also successfully discriminated MSA-P from MSA-C in the identification groups (MSA-C vs. MSA-P, p = .014, Supplementary Figure S1). The MSA subtype difference (MSA-P vs. MSA-C) pattern was characterized mainly by a relative metabolic increase in the cerebellum with some hypometabolism noted in the putamen and frontal region. Prospective TPR analysis confirmed pattern stability and specificity. MSA-P test subjects did not differ significantly from the MSA-P identification group (p = .821) but differed from both the MSA-C identification (p < .001) and test groups (p < .001). Similarly, MSA-C test subjects did not differ from the MSA-C identification group (p = .461) but differed from both MSA-P identification (p < .001) and test (p < .001) groups. These results indicate that the pattern robustly differentiated MSA-P and MSA-C in independent cohorts.

### Differences in relative regional metabolism

**Figure 3.**
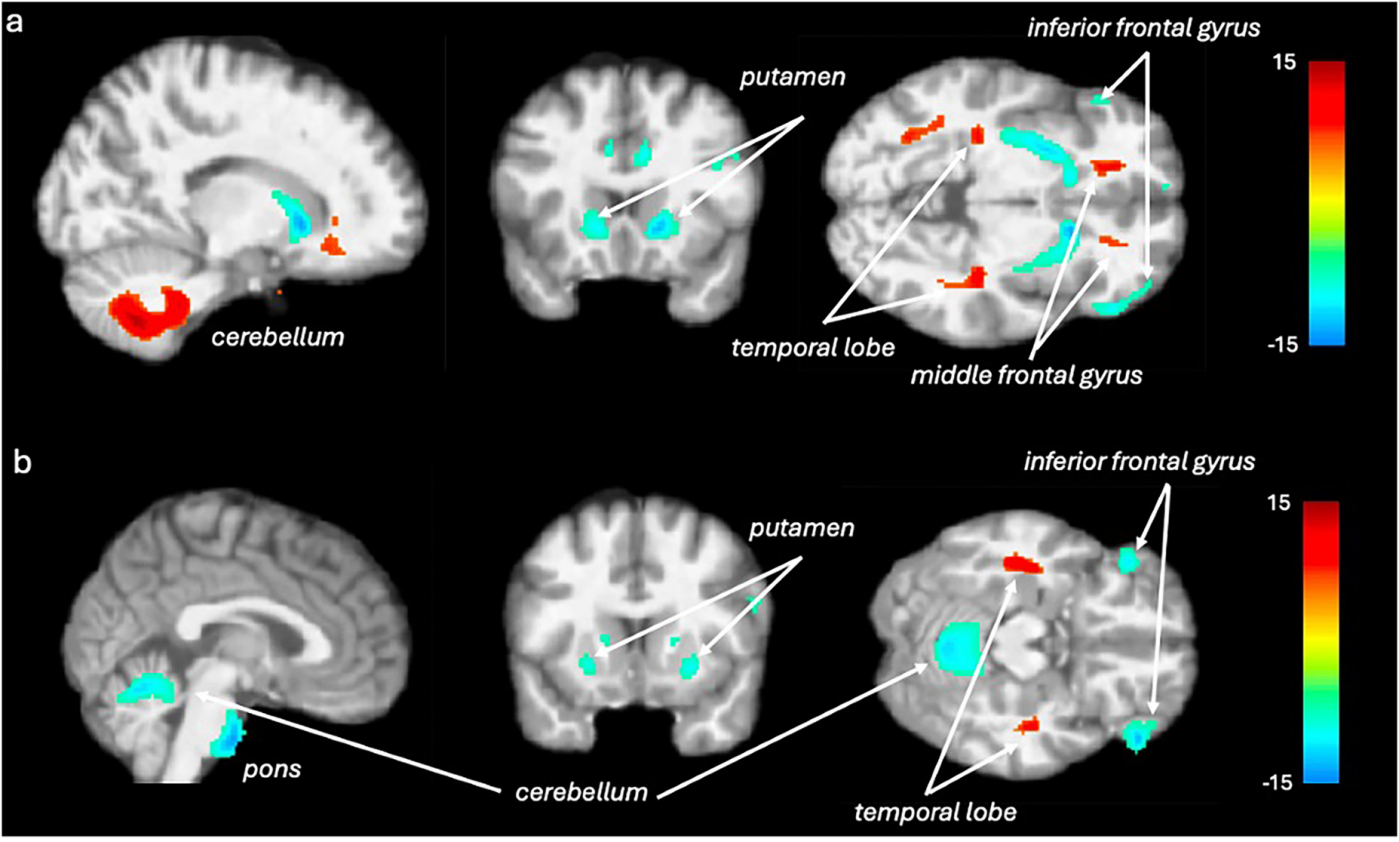
Brain regions with significant metabolic differences in MSA patients compared with healthy controls detected by SPM analysis of FDG-PET scans. a) Normalized glucose metabolism in the MSA-P patients decreased (blue) in the bilateral putamen, inferior frontal gyrus and anterior cingulate, and increased (red) in the bilateral temporal lobe, middle frontal gyrus and posterior lobe of the cerebellum compared to healthy controls (*p* < .001 uncorrected at peak-level). b) Normalized glucose metabolism in the MSA-C patients decreased (blue) in the vermis of the cerebellum, the pons, the bilateral inferior frontal gyrus, and the putamen, but increased (red) in the left and right temporal lobe compared to healthy controls (*p* < .001 uncorrected at peak-level). Only the clusters that are significant (p < .05) after FWE-correction are visualized.

SPM analysis of FDG-PET scans revealed bilaterally abnormal metabolism in the MSA patients compared to the healthy subjects. In the MSA-P group, relative regional metabolism decreased in the bilateral putamen, inferior frontal gyrus and anterior cingulate, and increased in the bilateral temporal lobe, middle frontal gyrus and posterior lobe of the cerebellum compared to healthy controls (*p* < .001; Figure 3A). In the MSA-C group, relative regional metabolism decreased in the vermis of the cerebellum, the pons, the bilateral inferior frontal gyrus, and the putamen, but increased in the left and right temporal lobe compared to healthy controls (*p* < .001; Figure 3B).

### Dopaminergic integrity

Significant differences in putamen DAT binding were observed across groups, *F*(2, 285) = 65.479, *p* < .001 (Figure 2C). MSA-P patients showed significantly reduced putamen DAT binding compared to healthy controls (*p* < .001) and the MSA-C group (*p* < .001), but no reduction was found in the MSA-C group compared to healthy controls (*p* = .989). Significant group differences were also seen for caudate DAT binding, *F*(2, 285) = 15.085, *p* < .001. In the caudate, DAT binding was reduced in MSA-P compared to MSA-C patients (*p* < .001), but no differences were found between healthy controls and MSA groups. These reductions remained significant following adjustments for age and sex. While no significant sex-differences in putamen DAT binding were found, significant differences in caudate DAT binding showed reductions in males compared to females in both MSA-P (*p* = .008) and MSA-C (*p* = .001) groups (Supplementary Table S2).

### Relationship between metabolic pattern expression and dopaminergic integrity

Significant correlations were found between MSAPRP-PC2 and DAT binding in both the caudate and putamen for the MSA-P group (Figure 2D). No significant correlations were observed between MSAPRP-Combined or MSAPRP-PC1 and striatal DAT binding in the MSA-P group or between MSACRP and striatal DAT binding in the MSA-C group (Supplementary Table S8).

### Clinical correlates

**Figure 4.**
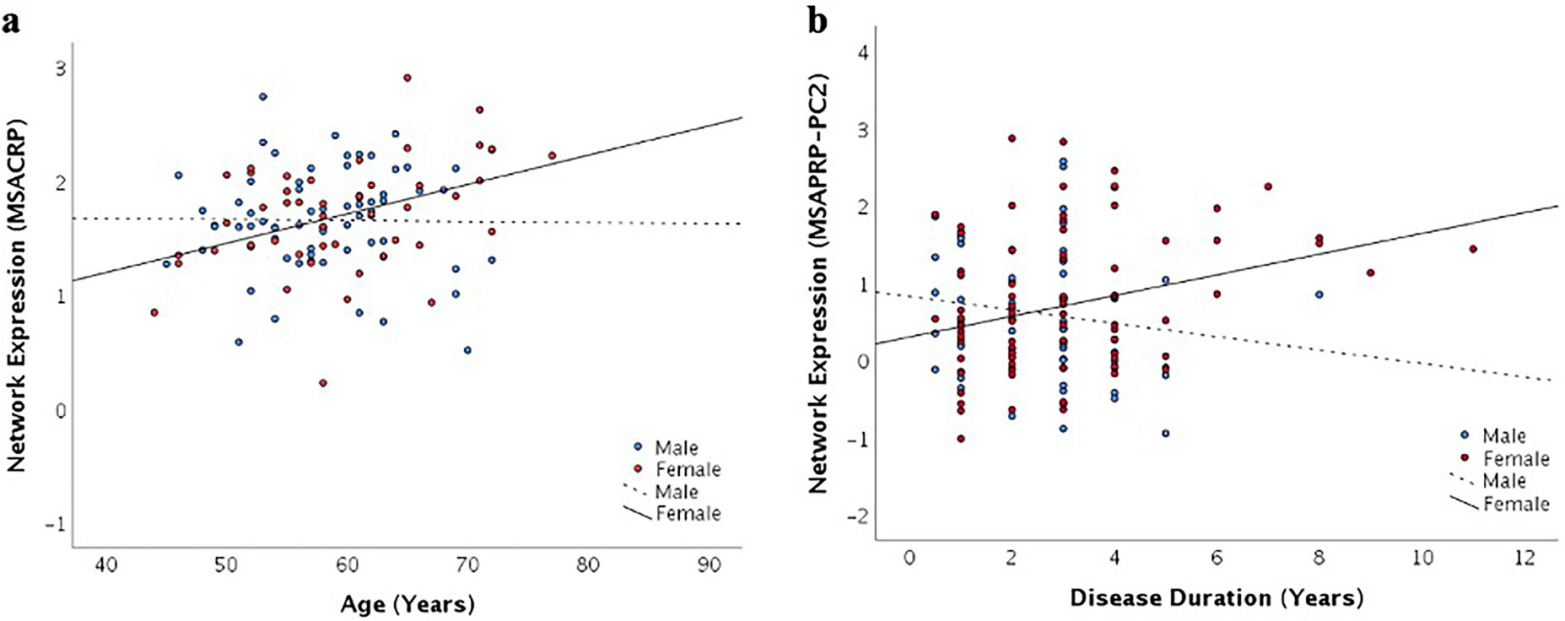
Sex-dependent relationship between clinical correlates and pattern expression score. a) Within the MSA-C group a significant sex-based interaction was found (*F*(1111) = 5.999, *p* = .016) and post-hoc analyses showed a significant correlation between age and MSACRP only for females (*r* = .393, *p* = .002). b) Within the MSA-P group a significant sex-based interaction was found (*F*(1135) = 7.667, *p* = .006), and post-hoc testing revealed a correlation between disease duration and MSAPRP-PC2 only for MSA-P females (*r* = .307, *p* = .005).

**Age.** In the MSA-P group, age correlated significantly with MSAPRP-Combined expression (*r* = .224, *p* = .007), MSAPRP-PC1 expression (*r* = .301, *p* = .001) and DAT binding in the putamen (*r* = .236, *p* = .005). A trend-level sex-based interaction was found when caudate DAT binding was considered, *F*(1135) = 3.707, *p* = .056, and post-hoc correlation analyses showed a significant association between age and caudate DAT binding for males (*r* = .343, *p* = .008; Figure 4A and Table 4).

**Table 4.** Correlations between network pattern expression scores / SUR values and age / disease duration.

		Age				Disease duration			
MSA-P	Pattern Expression	All	Sex-based Interaction	Male	Female	All	Sex-based Interaction	Male	Female
	MSARP-Combined	.224 (p = .007)	.128 (p = .721)	.241 (p = .066)	.214 (p = .052)	.016 (p = .848)	.638 (p = .426)	.15 (p = .258)	−.042 (p = .707)
	MSAPRP-PC1	.301 (p = .001)	.043 (p = .836)	.289 (p = .026)	.308 (p = .005)	−.089 (p = .291)	5.041 (p = .026)	.222 (p = .091)	−.216 (p = .05)
	MSAPRP-PC2	−.181 (p = .043)	.307 (p = .581)	−.144 (p = .277)	−.243 (p = .027)	.16 (p = .058)	7.667 (p = .006)	−.15 (p = .257)	.307 (p = .005)
	MSACRP	−.08 (p = .345)	.001 (p = .988)	−.069 (p = .604)	−.08 (p = .471)	.112 (p = .183)	2.601 (p = .109)	−.073 (p = .582)	.181 (p = .102)
	DAT Binding								
	Caudate	.176 (p = .037)	3.707 (p = .056)	.343 (p = .008)	.148(p = .180)	−.169 (p = .045)	1.701 (p = .194)	.039 (p = .77)	−.268 (p = .014)
	Putamen	.236 (p = .005)	.926 (p = .338)	.292 (p = .026)	.222 (p = .044)	−.246 (p = .003)	.79 (p = .376)	−.115 (p = .389)	−.305 (p = .005)
MSA-C	Pattern Expression								
	MSARP-Combined	.313 (p < .001)	2.128 (p = .147)	.212 (p = .07)	.422 (p = .001)	.067 (p = .445)	.695 (p = .406)	−.148 (p = .208)	−.013 (p = .923)
	MSAPRP-PC1	.255 (p = .003)	.001 (p = .983)	.268 (p = .021)	.24 (p = .072)	−.171 (p = .051)	.551 (p = .459)	−.126 (p = .286)	−.221 (p = .098)
	MSAPRP-PC2	.032 (p = .713)	3.591 (p = .06)	−.124 (p = .291)	.203 (p = .13)	.390 (p < .001)	.005 (p = .942)	.44 (p < .001)	.348 (p = .008)
	MSACRP	.190 (p = .03)	5.999 (p = .016)	−.006 (p = .958)	.393 (p = .002)	.227 (p = .009)	1.057 (p = .306)	.336 (p = .003)	.116 (p = .390)
	DAT Binding								
	Caudate	−.086 (p = .331)	.146 (p = .703)	−.061 (p = .604)	−.139 (p = .303)	−.027 (p = .757)	2.083 (p = .151)	−.190 (p = .105)	−.066 (p = .628)
	Putamen	−.003 (p = .975)	.057 (p = .812)	.016 (p = .896)	−.027 (p = .839)	−.087 (p = .322)	.952 (p = .331)	−.191 (p = .104)	−.018 (p = .894)

Values show the Pearson correlation coefficient (r) and the p-value. For the sex-based interactions, values show the F-test and the p-value. Bold values indicate significant based on Bonferroni correction for multiple comparisons.

In MSA-C, age also correlated significantly with MSAPRP-Combined expression (*r* = .313, *p* < .001) and MSAPRP-PC1 expression (*r* = .255, *p* = .003), but no other MSA-related pattern scores nor DAT binding when both sexes were considered. However a significant interaction effect was observed between sex and MSACRP scores predicting age, *F*(1111) = 5.999, *p* = .016. Post-hoc analyses revealed that the correlation between age and MSACRP only exists in females (*r* = .393, *p* = .002) but not in males (*r* = −.006, *p* = .958).

**Disease Duration.** In MSA-P, none of the MSA-related pattern scores were correlated with disease duration, however putamen DAT binding was (*r* = −.246, *p* = .003). A sex-based analysis showed a significant interaction effect with MSAPRP-PC2, *F*(1135) = 7.667, *p* = .006. Post-hoc analyses revealed a significant correlation between disease duration and MSAPRP-PC2 (*r* = .307, *p* = .005) only in females but not in males (*r* = −.15, *p* = 0.257; Figure 4B and Table 4).

In the MSA-C group, disease duration correlated with MSACRP (*r* = .227, *p* = .009) as well as with MSAPRP-PC2 (*r* = .39, *p* < .001), which was topographically similar to MSACRP. No significant interaction effect was observed with sex. DAT binding was not correlated with disease duration in MSA-C.

## Discussion

This study confirms the generalizability of previously characterized spatial covariance patterns in MSA, examining the relationship between metabolic activity and DAT binding in both MSA-P and MSA-C subtypes. In MSA-P patients, two significant sub-patterns emerged: one (MSAPRP-PC1) similar to a classic Parkinson's disease-related pattern (PDRP),^11,39^ with relative metabolic increases in the pons and posterior lobe of the cerebellum, pallidum and sensorimotor cortex that covaried with metabolic decreases in the lateral premotor cortex and parieto-occipital association regions, but hypometabolism in the striatum – the opposite of that found in the PDRP; and another (MSAPRP-PC2) characterized by metabolic reductions in the vermis of the cerebellum.^12,13,40^ When combined together, the overall MSAPRP (MSAPRP-Combined) showed relative metabolic increases in the pons, the posterior lobe of the cerebellum, the pallidum and in the sensorimotor cortex, which covaried with metabolic decreases in the caudate, putamen, the vermis of the cerebellum, the lateral premotor cortex and the parieto-occipital association regions. Topographical correlations indicated that this combined pattern was predominantly constituted of MSAPRP-PC1, with 72% spatial overlap compared to 27% spatial overlap with MSAPRP-PC2. It is not surprising, then, that MSAPRP-Combined and MSAPRP-PC1 showed similar results when examined with respect to DAT binding, age and disease duration.

In MSA-C, only one pattern emerged, characterized by relative metabolic reductions in both the putamen and vermis of the cerebellum. When compared to healthy controls, results were consistent showing increased metabolism in the cerebellum for the MSA-P group and decreased metabolism in the MSA-C group, with both groups showing decreased metabolism in the striatum. The consistent hypometabolic putamen found in both MSARPs strikingly contrasts to the striatal hypermetabolism found in PD, serving as an important discriminating factor in diagnosing MSA vs. PD, particularly for the MSA-P subgroup, which manifests in a similar manner to PD.

The MSACRP was topographically similar to MSAPRP-Combined. In addition, while the order was the opposite, MSACRP was also topographically overlapping with both MSAPRP-PC1 and -PC2. This suggests that MSA-C and MSA-P may be on the opposite end of continuous spectrum, represented by varying gradients of mixture of two orthogonal spatial patterns. To further test this hypothesis, we performed an additional SSM/PCA analysis directly comparing MSA-P and MSA-C patients without including healthy controls. This approach allowed identifying the specific pattern that differentiates the MSA subtypes. The resulting pattern reliably discriminated between MSA-P and MSA-C patients, and prospective TPR validation in independent test groups confirmed that the pattern is stable and specific to each subtype. Notably, MSA-P showed relative hypermetabolism in the cerebellum compared to MSA-C, suggesting it as a key brain region in differentiating MSA-P from MSA-C. Taken together, these findings provide additional evidence that MSA-P and MSA-C lie along a pathological spectrum, with stable and separable topographies reflecting their distinct disease expression. It is also noteworthy that MSA-P patients’ spatial metabolic patterns are consistently divided into two orthogonal patterns (PC1 and PC2) while MSA-C patients had only one pattern, which may represent that the two distinct pathologies (PD-like vs. cerebellar degeneration) can readily be dissociated within MSA-P while they were more inter-related within our MSA-C cohort.

It is important to acknowledge, however, that our findings may be influenced by the methodological choices used to define significant components. In this study, we included all principal components with a variance account for (VAF) of >5% and determined their significance using independent t-tests. Alternative strategies for component selection could yield different results. For example, another approach is to take the top PCs accounting for a cumulative 50% of the VAF and to use those PCs to compute Akaike Information Criterion (AIC) values, identifying the model with the best fit for distinguishing between groups.^33^ Studies employing this method have reported varying numbers of components used to define disease-related patterns; for instance, one study of MSA-C patients identified three components to define the MSACRP,^41^ while another study defined the MSAPRP using four components and the MSACRP using two.^19^ Such variability underscores how model-based selection strategies may emphasize different aspects of the data and potentially lead to the identification of different or additional spatial patterns.

As has been demonstrated in previous studies,^12,42–44^ FP-CIT DAT study results confirmed that MSA-C patients had relatively preserved striatal DAT binding, while MSA-P patients had significantly reduced putamen DAT binding compared to healthy controls and MSA-C. While caudate DAT binding in MSA-P was reduced compared to MSA-C, this reduction did not differ significantly from the healthy control group. Several previous studies have demonstrated lower overall striatal DAT binding in MSA-P than MSA-C, with relative sparing of the striatum in MSA-C,^12,45,46^ suggesting that degeneration of nigrostriatal neurons is a key feature of MSA-P.

Sex-based analysis revealed that males had significantly reduced DAT binding than females in the caudate, but not the putamen, across both MSA groups. Previous studies indicate higher striatal DAT activity in females than in males, both in healthy individuals and in patients with Parkinson's disease,^47–49^ particularly in the caudate.^48,49^ Female sex hormones (i.e., estrogen) are thought to play an important role in these sex-differences, suggesting a beneficial influence of estrogens against development and progression of Parkinson's disease.^50^ This study extends these findings to MSA, which presents as an atypical Parkinsonism.

In this study, DAT binding increased with age even after controlling for disease duration, contrary to the typical decrease seen with aging.^48^ This may suggest that earlier diagnosis of MSA is associated with more severe degeneration of nigrostriatal projections in both the caudate and putamen. This aligns with previous research showing early-onset MSA is associated with posterior-dominant decline in DAT binding in the putamen.^42^

A significant positive correlation was found between age and MSAPRP-Combined (and MSAPRP-PC1) in individuals with MSA-P even after controlling for disease duration, a finding consistent with previous research in PD.^51^ Age is one of the most significant risk factors for MSA and many other neurodegenerative disorders, and this finding suggests that age continues to influence brain metabolic patterns in MSA-P even after receiving a diagnosis. This correlation between age and MSAPRP-Combined was also present in MSA-C, further highlighting the common effects of aging in both subtypes of MSA.

In MSA-C, however, MSACRP itself was not significantly correlated with age after Bonferroni correction (*p* = .031), but a significant interaction effect was noted with sex. Post-hoc analysis revealed the age-MSACRP correlation was only present in females, persisting even after controlling for disease duration. It is not clear why there was no significant correlations in males.

Reduced DAT binding in the putamen correlated with increased disease duration in the MSA-P group, indicating more striatal degeneration as the disease progresses. Prior work links Parkinson's-like symptoms in MSA to postsynaptic striatal degeneration, causing hypoactivity in that region.^52^ Thus, disease progression leads to more severe degeneration and reduced DAT binding.

No significant correlation was found between disease duration and MSARPs in the overall MSA-P group. However, in females, disease duration positively correlated with increased MSAPRP-PC2 expression. While sex-based differences in MSA have not been extensively researched, previous PD studies have shown more pronounced structural^53^ and functional^47,54^ changes in males. It is possible that male MSA-P patients reached a ceiling in MSAPRP-PC2 expression early in disease progression, obscuring its correlation with disease duration. Additionally, the observed sex-difference may be partially driven by the greater number of females (n = 82) compared to males (n = 58) in our MSA-P cohort, which could influence statistical power and sensitivity to detect such relationships.

In MSA-C, no significant sex-based interaction was found between disease duration and MSACRP, though a strong overall correlation existed. The correlation was even stronger when MSAPRP-PC2 expression was examined within MSA-C, highlighting the topographical similarity between MSAPRP-PC2 and MSACRP. Both patterns were characterized by cerebellar hypometabolism, linking increased disease duration to decreased cerebellar metabolism. This aligns with the clinical observation that many MSA-P patients eventually exhibit cerebellar symptoms in later stages of disease progression.

In the MSA-P group, decreased DAT binding in the caudate and putamen correlated significantly with increased MSAPRP-PC2 expression, which showed hypometabolism in the putamen. This suggests that in MSA-P, presynaptic dopaminergic loss is associated with network-level changes encompassed by abnormal MSAPRP topography. In contrast, no such correlation was found in MSA-C despite elevated metabolic expression.

This study has several limitations. Misdiagnosis of MSA subtype is possible due to the lack of post-mortem confirmation,^55^ though MSA diagnostic criteria have a high diagnostic accuracy^56^ and a misdiagnosis of MSA is usually due to its confusion with Parkinson's disease or progressive supranuclear palsy, not the other way around.^55^ Also, partial-volume effects from PET scanner resolution and the sizes of the caudate and putamen cannot be excluded, though significant differences in DAT binding between MSA subtypes suggests that this issue likely did not affect the results.

It should be noted that the current study did not include detailed clinical measures such as the MSA rating scale (MSARS) or autonomic function indices, which limits the ability to directly link the identified metabolic network patterns to symptom severity. While we observed correlations of pattern expression and DAT binding with age, sex, and disease duration, these findings should be interpreted cautiously. By characterizing subtype-specific metabolic patterns in both MSA-P and MSA-C using SSM/PCA and prospectively assessing pattern expression in independent subject groups via TPR, our study demonstrates the reproducibility and stability of these networks and highlights their potential utility in differentiating MSA subtypes. Future work integrating metabolic and dopaminergic imaging with comprehensive clinical assessment of disease severity and autonomic function will be essential to clarify the clinical relevance of these imaging biomarkers.

In conclusion, metabolic imaging confirms the specificity of disease-related network topographies in MSA-P and MSA-C subtypes, while dopaminergic imaging provides complimentary insights into nigrostriatal degeneration. Considered together, metabolic pattern expression and DAT binding may help monitor disease progression in MSA subtypes. These measures also differ by sex, highlighting the importance of considering sex-based differences in understanding MSA subtypes. Future work to further elucidate sex differences will aid in understanding prognostic variations and support biomarker development and clinical trial design.

## Supplemental Material

sj-docx-1-pkn-10.1177_1877718X261434367 - Supplemental material for Different brain spatial metabolic patterns characterizing different subtypes of multiple system atrophy Supplemental material, sj-docx-1-pkn-10.1177_1877718X261434367 for Different brain spatial metabolic patterns characterizing different subtypes of multiple system atrophy by Tiffany Carther-Krone, Jarrad Perron, Chong Sik Lee and Ji Hyun Ko in Journal of Parkinson's Disease

## References

[1] Geser F Wenning GK Seppi K , et al. Progression of multiple system atrophy (MSA): a prospective natural history study by the European MSA study group (EMSA SG). Mov Disord 2006; 21: 179–186. 16161136 10.1002/mds.20678

[2] Quinn N . Multiple system atrophy: the nature of the beast revisited. Journal of neurology, neurosurgery and psychiatry 2020; 91: 1–4. 10.1136/jnnp-2018-318187 31848228

[3] Wenning GK Tison F ben Shlomo Y , et al. Multiple system atrophy: a review of 203 pathologically proven cases. Mov Disord 1997; 12: 133–147. 9087971 10.1002/mds.870120203

[4] Gilman S Low P Quinn N , et al. Consensus statement on the diagnosis of multiple system atrophy. Clin Auton Res 1998; 8: 359–362. 9869555 10.1007/BF02309628

[5] Gilman S Wenning GK Kaufmann H , et al. Second consensus statement on the diagnosis of multiple system atrophy. Neurology 2008; 71: 670–676. 18725592 10.1212/01.wnl.0000324625.00404.15 PMC2676993

[6] Iaccarino L Sala A Caminiti SP , et al. The emerging role of PET imaging in dementia [version 1; peer review: 3 approved]. F1000 Research 2017; 6: 1830–1830. 29071066 10.12688/f1000research.11603.1 PMC5639932

[7] Patel AB Lai JCK Chowdhury GMI , et al. Direct evidence for activity-dependent glucose phosphorylation in neurons with implications for the astrocyte-to-neuron lactate shuttle. Proceedings of the National Academy of Sciences - PNAS 2014; 111: 5385–5390. 10.1073/pnas.1403576111 PMC3986127 24706914

[8] Moeller JR Strother SC . A regional covariance approach to the analysis of functional patterns in positron emission tomographic data. J Cereb Blood Flow Metab 1991; 11: A121–A135. 10.1038/jcbfm.1991.47 1997480

[9] Spetsieris PG Eidelberg D . Scaled subprofile modeling of resting state imaging data in Parkinson's disease: methodological issues. NeuroImage (Orlando, Fla) 2011; 54: 2899–2914. 10.1016/j.neuroimage.2010.10.025 PMC3020239 20969965

[10] Eckert T Tang C Ma Y , et al. Abnormal metabolic networks in atypical parkinsonism. Mov Disord 2008; 23: 727–733. 18186116 10.1002/mds.21933

[11] Eidelberg D Moeller JR Dhawan V , et al. The metabolic topography of parkinsonism. J Cereb Blood Flow Metab 1994; 14: 783–801. 8063874 10.1038/jcbfm.1994.99

[12] Ko JH Lee CS Eidelberg D . Metabolic network expression in parkinsonism: clinical and dopaminergic correlations. J Cereb Blood Flow Metab 2017; 37: 683–693. 26980757 10.1177/0271678X16637880 PMC5381458

[13] Poston KL Tang CC Eckert T , et al. Network correlates of disease severity in multiple system atrophy. Neurology 2012; 78: 1237–1244. 20120404. 22491861 10.1212/WNL.0b013e318250d7fd PMC3324319

[14] Spetsieris PG Ma Y Dhawan V , et al. Differential diagnosis of parkinsonian syndromes using PCA-based functional imaging features. NeuroImage (Orlando, Fla) 2009; 45: 1241–1252. 10.1016/j.neuroimage.2008.12.063 19349238

[15] Tang CC Poston KL Eckert T , et al. Differential diagnosis of parkinsonism: a metabolic imaging study using pattern analysis. Lancet Neurol 2010; 9: 149–158. 20100108. 20061183 10.1016/S1474-4422(10)70002-8 PMC4617666

[16] Jin S Oh M Oh SJ , et al. Differential diagnosis of parkinsonism using dual-phase F-18 FP-CIT PET imaging. Nucl Med Mol Imaging 2013; 47: 44–51. 24895507 10.1007/s13139-012-0182-4 PMC4035208

[17] Shen B Wei S Ge J , et al. Reproducible metabolic topographies associated with multiple system atrophy: network and regional analyses in Chinese and American patient cohorts. Neuroimage Clin 2020; 28: 102416. 20200909. 32987300 10.1016/j.nicl.2020.102416 PMC7520431

[18] Teune LK Renken RJ Mudali D , et al. Validation of parkinsonian disease-related metabolic brain patterns. Mov Disord 2013; 28: 547–551. 23483593 10.1002/mds.25361

[19] Wu P Peng S Wang J , et al. 2017 International Congress of Parkinson's Disease and Movement Disorders. Vancouver, BC, June 4-8, 2017, pp.877. Hoboken, NJ: Wiley.

[20] Lee PH An Y-S Yong SW , et al. Cortical metabolic changes in the cerebellar variant of multiple system atrophy: a voxel-based FDG-PET study in 41 patients. NeuroImage (Orlando, Fla) 2008; 40: 796–801. 10.1016/j.neuroimage.2007.11.055 18203624

[21] Goebel G Seppi K Donnemiller E , et al. A novel computer-assisted image analysis of [123I]β-CIT SPECT images improves the diagnostic accuracy of parkinsonian disorders. Eur J Nucl Med Mol Imaging 2011; 38: 702–710. 21174092 10.1007/s00259-010-1681-0

[22] Nocker M Seppi K Donnemiller E , et al. Progression of dopamine transporter decline in patients with the Parkinson variant of multiple system atrophy: a voxel-based analysis of [123I]β-CIT SPECT. Eur J Nucl Med Mol Imaging 2012; 39: 1012–1020. 22460689 10.1007/s00259-012-2100-5

[23] Perju-Dumbrava LD Kovacs GG Pirker S , et al. Dopamine transporter imaging in autopsy-confirmed Parkinson's disease and multiple system atrophy. Mov Disord 2012; 27: 65–71. 22102521 10.1002/mds.24000

[24] Oh M Kim JS Kim JY , et al. Subregional patterns of preferential striatal dopamine transporter loss differ in Parkinson disease, progressive supranuclear palsy, and multiple-system atrophy. J Nucl Med 2012; 53: 399–406. 22323779 10.2967/jnumed.111.095224

[25] Kim HW Kim JS Oh M , et al. Different loss of dopamine transporter according to subtype of multiple system atrophy. Eur J Nucl Med Mol Imaging 2016; 43: 517–525. 26384682 10.1007/s00259-015-3191-6

[26] O'Sullivan SS Massey LA Williams DR , et al. Clinical outcomes of progressive supranuclear palsy and multiple system atrophy. Brain 2008; 131: 1362–1372. 20080402. 18385183 10.1093/brain/awn065

[27] Coon EA Nelson RM Sletten DM , et al. Sex and gender influence symptom manifestation and survival in multiple system atrophy. Auton Neurosci 2019; 219: 49–52. 20190424. 31122601 10.1016/j.autneu.2019.04.002 PMC6684211

[28] Watanabe H Saito Y Terao S , et al. Progression and prognosis in multiple system atrophy: an analysis of 230 Japanese patients. Brain 2002; 125: 1070–1083. 11960896 10.1093/brain/awf117

[29] Lu J Wang M Wu P , et al. Adjustment for the age- and gender-related metabolic changes improves the differential diagnosis of parkinsonism. Phenomics (Cham, Switzerland) 2023; 3: 50–63. 36939769 10.1007/s43657-022-00079-6 PMC9883378

[30] Gilman S Wenning GK Low PA , et al. Second consensus statement on the diagnosis of multiple system atrophy. Neurology 2008; 71: 670–676. 18725592 10.1212/01.wnl.0000324625.00404.15 PMC2676993

[31] Ashburner J Friston KJ . Diffeomorphic registration using geodesic shooting and Gauss–Newton optimisation. NeuroImage (Orlando, Fla) 2011; 55: 954–967. 10.1016/j.neuroimage.2010.12.049 PMC3221052 21216294

[32] Gaser C Dahnke R Thompson PM , et al. CAT: a computational anatomy toolbox for the analysis of structural MRI data. Gigascience 2024; 13: 1. 10.1093/gigascience/giae049 PMC11299546 39102518

[33] Spetsieris P Ma Y Peng S , et al. Identification of disease-related spatial covariance patterns using neuroimaging data. J Visualized Exp 2013; 76: e50319. 10.3791/50319 PMC3728991 23851955

[34] Ma Y Tang C Spetsieris PG , et al. Abnormal metabolic network activity in Parkinson's disease: test-retest reproducibility. J Cereb Blood Flow Metab 2007; 27: 597–605. 20060628. 16804550 10.1038/sj.jcbfm.9600358 PMC4455600

[35] Peng S Ma Y Spetsieris PG , et al. Characterization of disease-related covariance topographies with SSMPCA toolbox: effects of spatial normalization and PET scanners. Hum Brain Mapp 2014; 35: 1801–1814. 20130514. 23671030 10.1002/hbm.22295 PMC3778048

[36] Habeck C Foster NL Perneczky R , et al. Multivariate and univariate neuroimaging biomarkers of Alzheimer's disease. NeuroImage (Orlando, Fla) 2008; 40: 1503–1515. 10.1016/j.neuroimage.2008.01.056 PMC2441445 18343688

[37] Ko JH Spetsieris P Ma Y , et al. Quantifying significance of topographical similarities of disease-related brain metabolic patterns. PLoS One 2014; 9: e88119. 20140131. 10.1371/journal.pone.0088119 PMC3909315 24498250

[38] Rolls ET Huang C-C Lin C-P , et al. Automated anatomical labelling atlas 3. NeuroImage (Orlando, Fla) 2020; 206: 116189–116189. 10.1016/j.neuroimage.2019.116189 31521825

[39] Eidelberg D Moeller JR Fazzini E , et al. Metabolic correlates of pallidal neuronal activity in Parkinson's disease. Brain (London, England : 1878) 1997; 120: 1315–1324. 10.1093/brain/120.8.1315 9278625

[40] Tomše P Rebec E Studen A , et al. Abnormal metabolic covariance patterns associated with multiple system atrophy and progressive supranuclear palsy. Phys Med 2022; 98: 131–138. 20220507. 35537328 10.1016/j.ejmp.2022.04.016

[41] Ge J Peng S Wu P , et al. MRI-based Brain Networks of Perfusion and Volume in Cerebellar Variant of Multiple System Atrophy.

[42] Lee R Shin JH Choi H , et al. Variability of FP-CIT PET patterns associated with clinical features of multiple system atrophy. Neurology 2021; 96: e1663–e1671. 20210203. 10.1212/WNL.0000000000011634 33536274

[43] Bu LL Liu FT Jiang CF , et al. Patterns of dopamine transporter imaging in subtypes of multiple system atrophy. Acta Neurol Scand 2018; 138: 170–176. 20180324. 29573392 10.1111/ane.12932

[44] Kim HW Kim JS Oh M , et al. Different loss of dopamine transporter according to subtype of multiple system atrophy. Eur J Nucl Med Mol Imaging 2016; 43: 517–525. 20150919. 26384682 10.1007/s00259-015-3191-6

[45] Joling M Vriend C van den Heuvel OA , et al. Analysis of Extrastriatal. J Nucl Med 2017; 58: 1117–1123. 20161117. 27856628 10.2967/jnumed.116.182139

[46] Nicastro N Garibotto V Burkhard PR . 123I-FP-CIT SPECT accurately distinguishes parkinsonian from cerebellar variant of multiple system atrophy. Clin Nucl Med 2018; 43: e33–e36. 10.1097/RLU.0000000000001899 29215405

[47] Haaxma CA Bloem BR Borm GF , et al. Gender differences in Parkinson’s disease. Journal of Neurology, Neurosurgery and Psychiatry 2007; 78: 819–824. 17098842 10.1136/jnnp.2006.103788 PMC2117736

[48] Kaasinen V Joutsa J Noponen T , et al. Effects of aging and gender on striatal and extrastriatal [123I]FP-CIT binding in Parkinson's disease. Neurobiol Aging 2015; 36: 1757–1763. 20150122. 25697414 10.1016/j.neurobiolaging.2015.01.016

[49] Lee CS Kim SJ Oh SJ , et al. Uneven age effects of [(18)F]FP-CIT binding in the striatum of Parkinson's disease. Ann Nucl Med 2014; 28: 874–879. 20140710. 25008292 10.1007/s12149-014-0882-1

[50] Bourque M Dluzen DE Di Paolo T . Neuroprotective actions of sex steroids in Parkinson's disease. Front Neuroendocrinol 2009; 30: 142–157. 20090503. 19410597 10.1016/j.yfrne.2009.04.014

[51] Holtbernd F Ma Y Peng S , et al. Dopaminergic correlates of metabolic network activity in Parkinson's disease. Hum Brain Mapp 2015; 36: 3575–3585. 26037537 10.1002/hbm.22863 PMC6869564

[52] Sawle GV Playford ED Brooks DJ , et al. Asymmetrical presynaptic and postsynaptic changes in the striatal dopamine projection in dopa naive parkinsonism - diagnostic implications of the D2 receptor status. Brain (London, England : 1878) 1993; 116: 853–867. 10.1093/brain/116.4.853 8353712

[53] Tremblay C Abbasi N Zeighami Y , et al. Sex effects on brain structure in de novo Parkinson's disease: a multimodal neuroimaging study. Brain (London, England : 1878) 2020; 143: 3052–3066. 10.1093/brain/awaa234 32980872

[54] De Micco R Esposito F Nardo F , et al. Sex-related pattern of intrinsic brain connectivity in drug-naïve Parkinson's disease patients. Mov Disord 2019; 34: 997–1005. 31180598 10.1002/mds.27725

[55] Litvan I Goetz CG Jankovic J , et al. What is the accuracy of the clinical diagnosis of multiple system atrophy? A clinicopathologic study. Arch Neurol 1997; 54: 937–944. 9267967 10.1001/archneur.1997.00550200007003

[56] Osaki Y Wenning GK Daniel SE , et al. Do published criteria improve clinical diagnostic accuracy in multiple system atrophy? Neurology 2002; 59: 1486–1491. 12455559 10.1212/01.wnl.0000028690.15001.00

