# Supplementary material for: Different brain spatial metabolic patterns characterizing different subtypes of multiple system atrophy
Source: J Parkinsons Dis. 2026 Apr 15:1877718X261434367. Online ahead of print. doi: 10.1177/1877718X261434367 (PMC13435175; doi:10.1177/1877718X261434367)
Supplement: Supplementary file 1 [file sj-docx-1-pkn-10.1177_1877718X261434367.docx]

**Supplementary Results**

*Metabolic Network Expression*

Within the identification set, group differences between healthy controls and MSA-P patients were found for principal components 1 and 2. No differences were found for principal component 3. Group differences between healthy controls and MSA-C patients were found for principal component 1. No differences were found for principal components 2, 3 and 4 (Supplementary Table S1).

**Supplementary Table S1.** **Differences of subject scores in Principal Components between healthy controls and MSA patients in the identification set.**

Values are means ± standard deviation.

Analysis involving identification and test subjects together showed no significant differences between males and females in metabolic pattern expression scores or standard uptake ratios for neither the MSA-P (n = 140) or MSA-C (n = 131) groups in the putamen. A significant difference between males and females was found in both MSA-P and MSA-C groups when standard uptake ratio was compared in the caudate (Supplementary Table S2).

**Supplementary Table S2. Sex-Based Differences in Overall Network Expression and Striatal DAT Binding (identification and test sets combined).**

Values are means ± standard deviation.

*Test of Reliability of Metabolic Network Expression*

**Demographic Comparisons**

Demographic data from all participants in the original and replication identification sets are summarized in Supplementary Table S3. For both MSA-P and MSA-C groups, no significant differences in age, disease duration or sex were found between original and both replication sets.

**Supplementary Table S3. Comparisons between original and replication identification sets with respect to age, disease duration and sex.**

***Indicates inclusion of healthy control group in statistical analysis.**

**Age- and Sex-Matched Sample (The First Replication Set)**

To test the replicability of the originally defined MSARPs, new age- and sex-matched MSA-P and MSA-C identification groups were defined using the same procedure as outlined in the methods. No subjects were included in the replication sets that were in the original identification sets.

As outlined in the methods, only those components that accounted for at least 5% of subject-by-voxel variance were considered in identifying an MSA-related patterns. For MSA-P patients, the first four principal components (PCs) accounted for 45.03% of the subject-by-voxel variance, with PC1, PC2, PC3 and PC4 accounting for 22.19%, 11.18%, 6.27% and 5.39% respectively. Of these four PCs, the first two PCs significantly discriminated between MSA-P patients and healthy controls (Supplementary Table S4).

**Supplementary Table S4.** **Differences in Principal Components between healthy controls and MSA-P patients (the first MSA-P replication set).**

|  | **HC subjects** | **MSA-P patients** | **Significance** |
| --- | --- | --- | --- |
|  | **(n = 16)** | **(n = 16)** |  |
| **PC1** | .0001 ± .301 | 1.168 ± 1.12 | *t* = -4.035, *p* < .001 |
| **PC2** | -.001 ± .502 | 1.382 ± .892 | *t* = -5.402, *p* < .001 |
| **PC3** | .0001 ± 1.084 | .288 ± .921 | *t* = -.809, *p* = .425 |
| **PC4** | 0 ± .898 | .259 ± 1.106 | *t* = -.727, *p* = .473 |

Group differences between healthy controls and MSA-P patients were found for principal components 1 and 2. No differences were found for principal components 3 or 4. Values are means ± standard deviation.

For MSA-C patients, the first four PCs accounted for 45.63% of the subject by voxel variance, with PC1, PC2, PC3 and PC4 accounting for 19.98%, 11.61%, 7.63%, and 6.42%, respectively. Of these four PCs, both the first and third PCs significantly discriminated between MSA-C patients and healthy controls (Supplementary Table S5).

**Supplementary Table S5.** **Differences in Principal Components between healthy controls and MSA-C patients (the first MSA-C replication set)**.

|  | **HC subjects** | **MSA-C patients** | **Significance** |
| --- | --- | --- | --- |
|  | **(n = 16)** | **(n = 16)** |  |
| **PC1** | 0 ± .346 | 1.53 ± .835 | *t* = -6.768, *p* < .001 |
| **PC2** | 0 ± .371 | .052 ± 1.388 | *t* = -.144, *p* = .887 |
| **PC3** | -0.0000625 ± .444 | .875 ± 1.209 | *t* = -2.719, *p* = .011 |
| **PC4** | 0.0000625 ± .976 | .327 ± 1.029 | *t* = -.922, *p* = .364 |

Group differences between healthy controls and MSA-C patients were found for principal components 1 and 3. No differences were found for principal components 2 and 4. Values are means ± standard deviation.

**Randomly Selected Sample (The Second Replication Set)**

As a second test of the replicability of the originally defined MSARPs, new MSA-P and MSA-C identification groups were defined by randomly selecting 16 participants from each group. Subjects who were included in the original identification set and the first replication set were excluded from this selection.

For MSA-P patients, the first five principal components (PCs) accounted for 49.22% of the subject-by-voxel variance, with PC1, PC2, PC3, PC4 and PC5 accounting for 21.51%, 9.02%, 7.38%, 6.25% and 5.06% respectively. Of these five PCs, the first two PCs significantly discriminated between MSA-P patients and healthy controls (Supplementary Table S6).

**Supplementary Table S6.** **Differences in Principal Components between healthy controls and the randomly selected MSA-P patients (the second MSA-P replication set).**

|  | **HC subjects** | **MSA-P patients** | **Significance** |
| --- | --- | --- | --- |
|  | **(n = 16)** | **(n = 16)** |  |
| **PC1** | .0001 ± .309 | 1.377 ± .98 | *t* = -5.361, *p* < .001 |
| **PC2** | 0 ± .457 | .999 ± 1.151 | *t* = -3.224, *p* = .003 |
| **PC3** | 0 ± .508 | .473 ± 1.3 | *t* = -1.357, *p* = .185 |
| **PC4** | -.0001 ± .799 | .154 ± 1.19 | *t* = -.429, *p* = .671 |
| **PC5** | .0001 ± .647 | .585 ± 1.211 | *t* = -1.704, *p* = .099 |

Group differences between healthy controls and MSA-P patients were found for principal components 1 and 2. No differences were found for principal components 3, 4 or 5. Values are means ± standard deviation.

For MSA-C patients, the first five principal components (PCs) accounted for 46.72% of the subject-by-voxel variance, with PC1, PC2, PC3, PC4 and PC5 accounting for 17.31%, 11.4%, 6.71%, 5.72% and 5.58% respectively. Of these five PCs, only the first PC significantly discriminated between MSA-C patients and healthy controls (Supplementary Table S7).

**Supplementary Table S7.** **Differences in Principal Components between healthy controls and the randomly selected MSA-C patients (the second MSA-C replication set)**.

|  | **HC subjects** | **MSA-C patients** | **Significance** |
| --- | --- | --- | --- |
|  | **(n = 16)** | **(n = 16)** |  |
| **PC1** | -.0000625 ± .348 | 1.669 ± .678 | *t* = -8.761, *p* < .001 |
| **PC2** | 0 ± .471 | .211 ± 1.349 | *t* = -.59, *p* = .560 |
| **PC3** | .000125 ± .895 | .357 ± 1.094 | *t* = -1.011, *p* = .320 |
| **PC4** | -.000125 ± .855 | .376 ± 1.123 | *t* = -1.066, *p* = .295 |
| **PC5** | -.0000625 ± .813 | .343 ± 1.158 | *t* = -.970, *p* = .340 |

Group differences between healthy controls and MSA-C patients were found for principal component 1. No differences were found for principal components 2, 3, 4 or 5. Values are means ± standard deviation.

*Relationship between Metabolic Pattern Expression and Dopaminergic Integrity*

Significant correlations were found between MSAPRP-PC2 and DAT binding in both the caudate and putamen for the MSA-P group. No significant correlations were observed between MSAPRP-PC1 and striatal DAT binding in the MSA-P group or between MSACRP and striatal DAT binding in the MSA-C group (Supplementary Table S8).

**Supplementary Table S8.** **Correlations between metabolic pattern expression scores and DAT binding (identification and test sets combined).**

Values show the Pearson correlation coefficient (*r*) and the p-value. Bold values indicate significant based on Bonferroni correction for multiple comparisons.

**Supplementary Figure S1.** **Subtype-specific comparison of topography and metabolic expression directly comparing MSA-P and MSA-C subgroups.** **a)** Metabolic patterns derived from the SSM/PCA analysis were masked with binary masks created from ICV maps threshold at z = 1.96. MSA-P showed relatively increased metabolism in the cerebellum and relatively decreased metabolism in the putamen and frontal area compared to MSA-C. **b)** SSMPCA was performed with MSA-C patients as the control group and MSA-P patients as the test group. The resulting pattern successfully discriminated between the two subtypes, with prospective TPR validation showing stable expression across independent identification and test groups. Pattern expression scores did not differ significantly between identification and test groups within each subtype but differed between subtypes. *Significantly different from MSA-C identification. ^Significantly different from MSA-C test.
